# Current challenges and implications for dengue, chikungunya and Zika seroprevalence studies worldwide: A scoping review

**DOI:** 10.1371/journal.pntd.0006533

**Published:** 2018-07-16

**Authors:** Camille Fritzell, Dominique Rousset, Antoine Adde, Mirdad Kazanji, Maria D. Van Kerkhove, Claude Flamand

**Affiliations:** 1 Epidemiology Unit, Institut Pasteur de la Guyane, Cayenne, French Guiana; 2 National Reference Laboratory for Arboviruses, Institut Pasteur de la Guyane, Cayenne, French Guiana; 3 Center for Global Health, Institut Pasteur de Paris, Paris, France; VIET NAM

## Abstract

**Background:**

Arboviral infections are a public health concern and an escalating problem worldwide. Estimating the burden of these diseases represents a major challenge that is complicated by the large number of unapparent infections, especially those of dengue fever. Serological surveys are thus required to identify the distribution of these diseases and measure their impact. Therefore, we undertook a scoping review of the literature to describe and summarize epidemiological practices, findings and insights related to seroprevalence studies of dengue, chikungunya and Zika virus, which have rapidly expanded across the globe in recent years.

**Methodology/Principal findings:**

Relevant studies were retrieved through a literature search of MEDLINE, WHOLIS, Lilacs, SciELO and Scopus (2000 to 2018). In total, 1389 publications were identified. Studies addressing the seroprevalence of dengue, chikungunya and/or Zika written in English or French and meeting the inclusion and exclusion criteria were included. In total, 147 studies were included, from which 185 data points were retrieved, as some studies used several different samples. Most of the studies were exclusively conducted on dengue (66.5%), but 16% were exclusively conducted on chikungunya, and 7 were exclusively conducted on Zika; the remainder were conducted on multiple arboviruses. A wide range of designs were applied, but most studies were conducted in the general population (39%) and in households (41%). Although several assays were used, enzyme-linked immunosorbent assays (ELISAs) were the predominant test used (77%). The temporal distribution of chikungunya studies followed the virus during its rapid expansion since 2004. The results revealed heterogeneity of arboviruses seroprevalence between continents and within a given country for dengue, chikungunya and Zika viruses, ranging from 0 to 100%, 76% and 73% respectively.

**Conclusions/Significance:**

Serological surveys provide the most direct measurement for defining the immunity landscape for infectious diseases, but the methodology remains difficult to implement. Overall, dengue, chikungunya and Zika serosurveys followed the expansion of these arboviruses, but there remain gaps in their geographic distribution. This review addresses the challenges for researchers regarding study design biases. Moreover, the development of reliable, rapid and affordable diagnosis tools represents a significant issue concerning the ability of seroprevalence surveys to differentiate infections when multiple viruses co-circulate.

## Author summary

Arthropod-borne viruses (arboviruses) are among the most important of the emerging infectious disease public health problems facing the world. The actual impact of arboviruses worldwide remains unknown, and estimating the true burden of these diseases represents a current challenge. Serological surveys are the most reliable tool for estimating the impact of arboviruses outbreaks in a given territory, and the results of such surveys have implications for potential mitigation measures such as vaccination. We undertook a thorough review of the literature produced from 2000 to March 15, 2018, addressing the seroprevalence of dengue, chikungunya and/or Zika to describe and summarize methodological approaches and map the geographical distribution of seroprevalence studies for these three viruses worldwide. A total of 185 studies addressing the seroprevalence of dengue, chikungunya and/or Zika were included in the review. Most of the studies were exclusively conducted on dengue (66.5%), but 16% were exclusively conducted on chikungunya, and 7 studies were exclusively conducted on Zika; the remainder were conducted on multiple arboviruses. Our study reveals that a wide range of methodological designs were applied regarding population, recruitment and/or laboratory testing. This study also highlights the high seroprevalence heterogeneity between continents and within a given country for dengue, chikungunya and Zika viruses. The results underscore existing gaps in seroprevalence studies distribution worldwide and the need to develop the most sensitive and specific diagnosis tool to provide recommendations for future serological studies.

## Introduction

### Background

Arboviral infections have become a significant public health problem with the emergence and re-emergence of arboviral diseases worldwide in recent decades. Arboviruses are considered emerging or re-emerging pathogens based on their geographic spread and increasing impact on susceptible populations. For instance, dengue virus (DENV) infection, once rare, is now estimated to be the most common arboviral infection globally, with transmission occurring in at least 128 countries and with nearly 4 billion people at risk [1,2]. Over the period 2000–2010, an unprecedented increase in the number of cases was reported in the Americas, circulating all four serotypes (DENV1-DENV2-DENV3-DENV4) and reaching the highest record of cases ever reported over a decade [3]. DENV is now hyperendemic in many parts of the tropics and subtropics. The recent emergence of chikungunya virus (CHIKV) in the Caribbean in 2013 and its rapid spread to 45 countries and territories in North, Central, and South America highlight its high potential for epidemics [4]. In the aftermath of this emergence, Zika virus (ZIKV) aroused global attention due to its rapid spread since its first detection in May 2015 in Brazil to 22 other countries and other territories in the Americas [5].

Given the increasing number of cases; geographic spread; and health, social and economic impact of arboviral outbreaks, estimating their true burden represents a crucial issue but remains a difficult task. In their acute stages, arboviral infections cause a broad spectrum of disease, ranging from asymptomatic infection to severe disease, which can lead to misclassification in case reporting, especially when several arboviruses co-circulate [6]. Furthermore, surveillance systems, which generally rely on clinicians, hospitals and laboratory reports, are appropriate for helping detect outbreaks promptly but are not designed to estimate the real disease burden and tend to underestimate the total number of cases. In fact, because of the nature of arboviral infections with 75%, between 3 and 25% and 80% of asymptomatic cases for DENV, CHIKV and ZIKV respectively [1,7,8] and because healthcare seeking can vary greatly based on access to care, surveillance data alone can be unreliable [9].

Accordingly, some studies have estimated the burden of DENV outbreaks using a range of empirical or extrapolative methods and disease-modeling approaches [1,10,11]. However, the most reliable data for empirical assessments are drawn from seroprevalence studies, which are often lacking. In fact, these seroprevalence surveys are expensive and difficult to perform; such surveys require important logistical resources, including a large workforce (e.g., supervisors, technicians, physicians, nurses or phlebotomists, epidemiologists, statisticians, and field investigators) and biological support (e.g., sufficient freezer space for sample storage and reagents and kits for testing). Moreover, establishing good and reliable tests for arboviruses is an important task for public health institutions, especially when symptoms are difficult to distinguish from other common febrile illnesses and when cross-reactivity is observed [12]. The problem of cross-reactivity, as a result of the co-circulation of multiple arboviruses belonging to the same family in the same area, requires additional tests and thereby increases overall cost, time and labor [13].

However, data on arboviruses prevalence rates are essential for understanding their geographical distribution as well as their contribution to global morbidity and mortality. Such information is critical for determining the optimal allocation of the limited resources available for disease control and evaluating the impact of prevention policies and strategies such as vaccination. The rationale for conducting serological studies is straightforward; these studies provide surveillance that complements traditional symptom-based and laboratory-based surveillance. Serological studies provide an alternative approach for monitoring immunity levels in a population and do not require that people be tested during the short period when they are symptomatic [14]. In our research, seroprevalence can be defined as the frequency of individuals in a given population presenting evidence of a prior infection based on serological tests or a combination of serological and virological tests.

### Populations and study design of the serological surveys

Seroprevalence studies can be conducted using multiple designs and among various populations involving a general population or specific or relevant population subgroups.

The general population concept is widely used in seroprevalence studies, but few studies provide a clear definition, and ambiguities related to the definition exist in the context of almost every country. Here, we present a definition that will be used throughout the review to classify serologic surveys according to the study population. A “general population” refers to the people (without any ethnic, socio-economic or health status restrictions) who inhabit a given area, usually in terms of political or geographical boundaries. The area may be quite small in size and population (e.g., a village of one hundred people) or quite large (e.g., a nation of one million people). A general population survey involves the collection of data to characterize all, or nearly all, people living in the area. Because of financial and logistical constraints, the data are typically collected from a representative sample of people residing in that area through a combination of personal interviews, administered on site using a standardized questionnaire, and blood samples drawn by skilled personnel (doctor, nurse or phlebotomist). Although surveys of the general population may gather data about inhabitants of all ages, lower and/or upper age limits are typically placed on eligible respondents, especially when blood samples are needed.

In contrast to general population surveys, specific population surveys focus on subgroups, (e.g., pregnant women, school children, blood donors, and patients). These subgroups are defined by membership in or contact with some social institution or by the presence of exposure. Furthermore, regardless of the type of population, because a census is resource-intensive, random sampling is highly recommended as a cost-effective method for obtaining seroprevalence estimates that are representative of the target population. Convenience sampling, such as selecting administrative units or schools that are easy to sample, is expected to result in bias. The reason is that administrative units selected because of convenience may not be generalizable to the larger population [9].

Seroprevalence studies can also use different designs, including cross-sectional, prospective, and retrospective designs, and can refer to cohort or case-control studies.

In the context of emerging and re-emerging arboviral diseases worldwide, we undertook a scoping review of the literature to describe and summarize the epidemiological practices, findings and insights related to seroprevalence studies reported worldwide over the recent period of 2000 to 2017, which was marked by an unprecedented increase in the number of arboviruses cases registered across the globe.

## Materials and method

A literature review group (CFr, CFl) developed the protocol for conducting this literature review based on the Preferred Reporting Items of Systematic Reviews and Meta-Analyses (PRISMA) statement.

### Search strategy

Screening was first conducted through an online MEDLINE (United States National Library of Medicine) search for English- or French-language literature published between January 2000 and March 2018. Between November 2016 and March 2018, we searched several electronic databases with reference to the expanded Medical Subject Headings (MeSH) thesaurus, using the following search terms: [“arbovirus” or “arbovirus infection” or “dengue” or “chikungunya” or “zika”] AND [“seroepidemiologic studies” or “seroprevalence” or “seroepidemiology” or “serosurvey”]. The databases included the following: MEDLINE, World Health Organization Library database (WHOLIS), Latin American and Caribbean Health Sciences Database (Lilacs), Scientific Electronic Library Online (SciELO) and Scopus. A free search was also conducted through the Google search engine. Additional studies were identified through manual searches of the reference lists of identified papers. No attempt was made to identify unpublished studies. After deleting duplicates, the literature review group systematically screened the title, abstract and full text of each study for the inclusion and exclusion criteria.

### Selection

Articles were excluded if (i) the studies were published before January 1, 2000, or after March 15, 2018; (ii) the studies were published in languages other than English or French; (iii) the study sample included febrile patients, hospitalized patients, suspected or confirmed cases, or HIV or malaria patients because they are likely to provide biased estimates of seroprevalence, as well as if the study sample included immigrants, military personnel, travelers, or relief workers; and (iv) they were prospective/retrospective cohort studies that did not provide a baseline seroprevalence, because these study designs are likely to be associated with a specific first objective that only rarely focuses on determining seroprevalence rates.

We included cross-sectional and cohort studies analyzing samples from the general population, pregnant women, blood donors, age-specific subgroups, healthy volunteers and school children as possible sources of information about arboviruses seroprevalence.

### Data extraction

Data from the selected sources were collated and summarized using a table consisting of a series of Excel spreadsheets. Eligible articles were abstracted for publication metadata, settings, design, population sampling approach, sample size, laboratory assays, age categories, seroprevalence rates, ethical approval and reported biases. When a study used several separate samples (e.g., from different countries or different study populations or age group), it was separated, and each sample was considered a unique data point. Duplicate citations were removed. When articles were not available or did not provide sufficient information, we contacted the authors for additional information.

## Results

### Literature search

**Fig 1:**
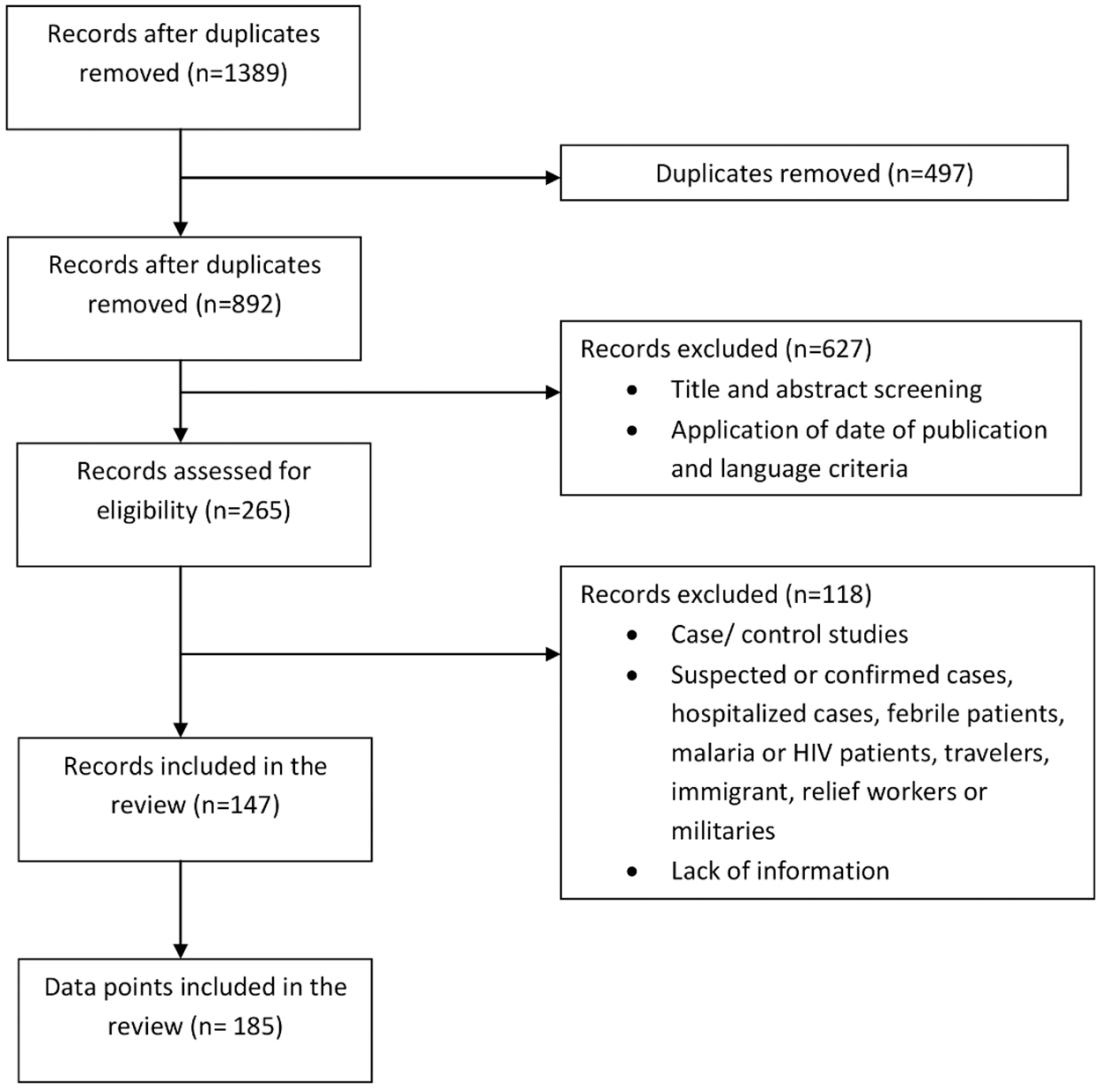
Flowchart of dengue, chikungunya and Zika seroprevalence studies used in the review.

We identified 265 unique studies reporting the seroprevalence of dengue, chikungunya or Zika that were eligible for full-text review (Fig 1). Among these studies, 18% (n = 48) were prospective or retrospective cohort or case-control studies, among which 16 studies provided a seroprevalence at baseline and enrolled participants according to our inclusion criteria. With respect to the study populations, 39.6% (n = 105) of these studies targeted febrile patients, hospitalized cases, suspected or confirmed cases, malaria or HIV patients, travelers, immigrants, relief workers or military personnel. Incomplete information was available for three studies. In total, 118 studies did not fulfill the inclusion criteria. Ultimately, the review was based on 185 data points from 147 unique studies (Fig 1). A description of the included studies is available in S1 Appendix.

The majority of the studies were exclusively conducted on dengue [15–112] (n = 123), with 16.% exclusively conducted on chikungunya (n = 29) [113–136] and 12% conducted on both dengue and chikungunya (n = 23) [137–154]; furthermore, seven studies were conducted on Zika [8,155–160], one study was conducted on both dengue and Zika [161] and two studies were conducted on both viruses [162].

### Survey methods used to measure seroprevalence

#### Ethical approval

Each article was reviewed to determine whether ethical approval was reported. Most studies obtained national ethics approval (58%), and 27% obtained international ethics approval from more than one country. Five studies reported not requiring institutional review board approval because they represented public health studies. Eleven studies did not provide indicative information about ethics approval but mentioned in their method section that mandatory written informed consent from each individual was obtained. Five studies provided no indicative information about ethics approval or written consent.

#### Target population

The population type distribution is presented in Table 1. We observed that most studies were conducted in a general population (38.9%), followed by studies conducted among age-specific subgroups (22.7%) and pregnant women (15.7%). Blood donors and schoolchildren were less well represented among the included studies (13.5% and 8.1%, respectively).

**Table 1:** Distribution of design features across studies (n = 185).

	N	Frequency (%)
**Population type**		
General population	72	38.9
Age-specific subgroups*	42	22.7
Pregnant women	29	15.7
Blood Donors	25	13.5
School children	15	8.1
Not specified	2	1.1
**Recruitment**		
Household	76	41.5
Hospital	44	24.0
Blood donor center	19	10.4
School	14	7.7
Previous studies	11	6.0
Center recruitment**	8	4.9
Laboratory	6	3.3
Not specified	4	2.0
**Sample size**		
<200	27	14.6
[200–499]	66	35.7
[500–999]	36	19.5
[1000–1999]	31	16.8
> = 2000	25	13.5
TOTAL	185	100

*Age-specific subgroups included individuals belonging to a specific age group (e.g., infants, children, adolescents or adults).

** Center recruitment included specific locations or centers with the aim of enrolling volunteers (e.g., a grocery store, a meeting place in a village, a mobile team or a university).

The target age groups selected for the serosurveys were variable. Mostly, studies were conducted among all age groups and adults (>15 years old) (38% and 34%, respectively). However, nearly 20% of the studies were conducted among infants, children or adolescents. For 17 studies conducted among pregnant women, age was not mentioned; thus, we named the age group “women of reproductive age”, and the others were classified into the “adolescent and adult” category. Relatively few studies focused on infants and children (10%), as it can be difficult to include these populations in surveys. Nevertheless, among studies conducted among all age groups, 78% provided age ranges, and 47% of these studies included individuals from 0 to 1 years old, 15% included individuals from 2 to 3 years old, and 38% included individuals from 4 to 5 years old. Finally, nearly half (42%) of these studies presented seroprevalence data stratified by age.

#### Design and sample size

We were able to categorize the studies according to sample recruitment for all studies except one (Table 1). Most studies were conducted through households (41.5%), and approximately one-quarter were conducted through hospital facilities. We noted that 89% of the studies conducted in a general population occurred in households, whereas the others recruited banked samples collected for routine check-ups or vaccinations in hospitals and laboratories or from previous studies.

The sample size for each study varied from 46 to 5669. For the analysis, the sample size was divided into 5 groups: <200; 200–499; 500–999; 1000–2000; and >2000 participants. The category that was most represented was [200–499] (35.7%). The mean sample size was significantly higher when the studies were conducted in blood centers (p<0.01). However, samples from blood banks are not considered random samples; people who donate blood are different from the general population.

Only 17% of the studies provided response rates, which ranged from 40 to 100% (mean of 80%). These rates did not differ significantly according to the population or the place of survey.

#### Blood collection and serological and molecular tests

With respect to blood collection, most studies collected blood through venous puncture (91.5%), and 8.5% collected blood using finger prick; one study used both techniques.

The review reported a wide range of assays used across studies. However, the main reported tests were the IgG enzyme-linked immunosorbent assay (ELISA), with more than half of the studies using the indirect method (77%), followed by the IgM ELISA (37.3%) and neutralization tests (plaque reduction neutralization tests (PRNTs) or microneutralization tests) (25.4%).

**Fig 2:**
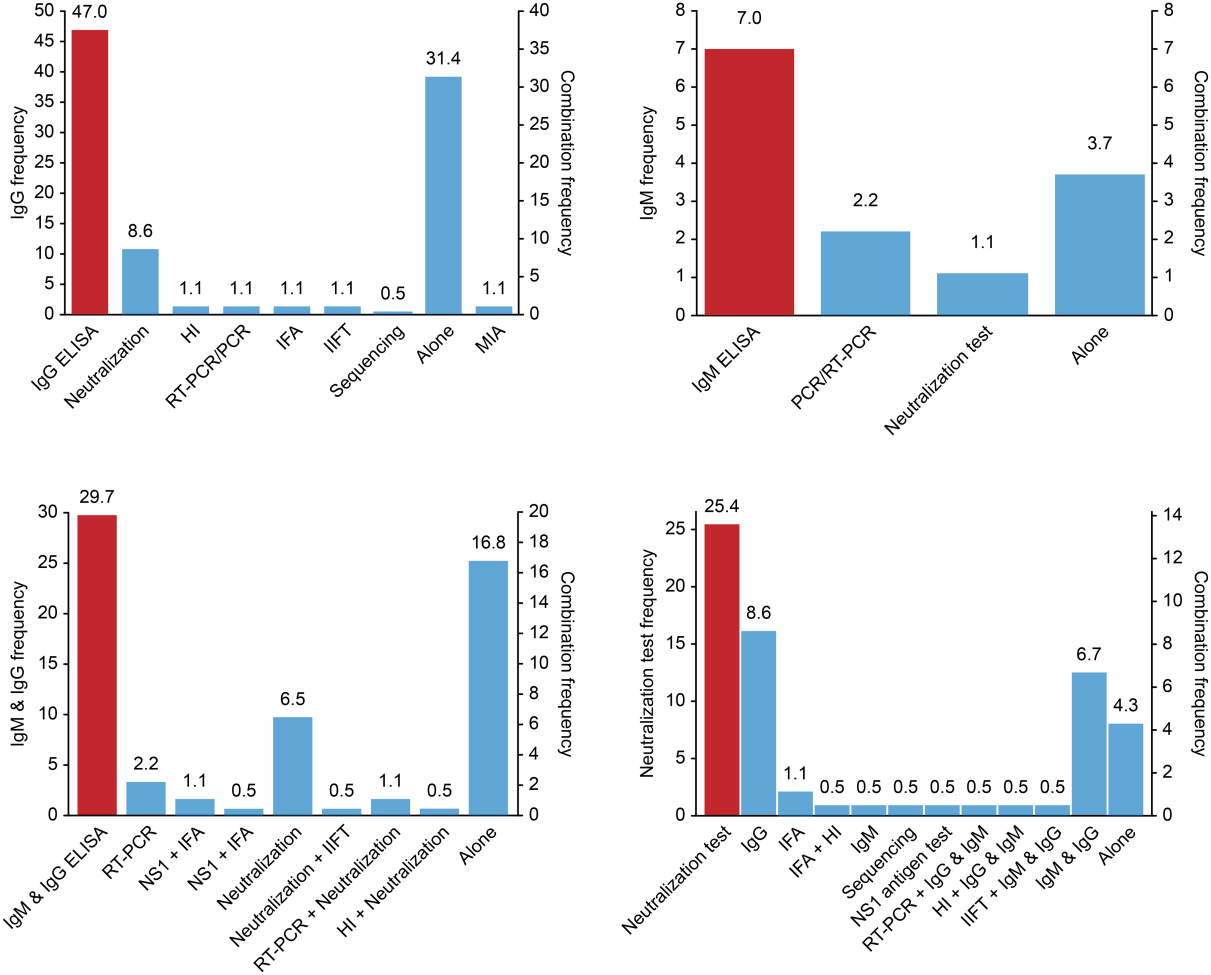
Distribution of laboratory test combinations across studies. HI: Hemagglutination inhibition; IFA: Immunofluorescence assay; IIFT: Indirect immunofluorescence test; PCR: Polymerase chain reaction; RT-PCR: Reverse transcription PCR; MIA: Microsphere immunoassays; NS1: Non-structural protein 1 antigen test.

As shown in Fig 2, most tests were combined with other methods, except IgG ELISA, which was performed alone in one-third of cases. All viral detection tests were associated with an immunoassay. We noted that the majority of the studies using IgM ELISA [8,33,35,36,38,44,54,56,60,68,71,76,82,87,108,119,120,120,121,125,128,130–133,139–141,143], NS1 tests [149] and/or RT-PCR [35,60,76,108,118,141,159,160] were conducted in an outbreak or post-outbreak context (p<0.001), which is not surprising because these tests are used for detecting acute and/or recent infections. Moreover, nearly all cohort studies that described the incidence and/or seroconversion of arboviral infection also used IgM ELISA and/or RT-PCR methods [98,103–105,107–109,111,160].

Results showed that virus neutralization assays are still widely used, despite their tedious nature (25.4%), as they can differentiate monotypic from multitypic dengue exposure. More than two-thirds of the studies that used neutralization tests performed this technique only on positive sera by serology to complete the results and to confirm the infecting flavivirus (for DENV and ZIKV).

#### Social studies and entomological surveys

Only three studies focused on social determinants of disease, assessing the knowledge, attitudes and practices of the population [63,70,122]. Nearly 10% of the studies set up an entomological survey. Among these studies, only three reported an association between entomological results and seroprevalence as well as the presence of breeding sites as predictors of seropositivity [38,54,57].

#### Estimation of asymptomatic infection

With respect to arboviruses, a wide variation in disease spectrum, including asymptomatic infection, is often observed. In the review, 43 studies reported the proportion of asymptomatic forms; these rates ranged between 22% and 99% for DENV (mean: 67%) [23,35,42–44,48,51,54,56,59,72,73,76,108,152,163], between 4% and 65% for CHIKV (mean: 26%) [119,120,122,123,125,126,128,129,131,133,141,149,152,163] and between 29% and 80% for ZIKV (mean: 55%) [8,155,159,160]. However, the rates did not differ significantly among continents for either arbovirus, irrespective of whether the highest rates were in the Americas. Moreover, we observed no differences between the asymptomatic rate and age group category for either infection.

#### Multiple infections

Approximately 9% of studies assessed the proportion of single and multiple DENV infections using neutralization tests, and one study performed both PRNT and NS1 serotype-specific IgG ELISA tests [15,29,31,36,37,48,66,77,82,93,95]. All studies performed serotyping on a subsample of seropositive sera except for three studies, which performed the test among all sera, ranging from 164 to 1151 individuals. Most studies reported an association between multitypic response and increasing age. The main reported limitation indicated that although it was clear that the population had been exposed to more than one DENV serotype, neutralization assays did not distinguish between homotypic and heterotypic dengue neutralization responses in case of sequential infections by various DENV serotypes.

#### Factors associated with seroprevalence

Several factors found to be associated with seroprevalence are presented in Table 2. Seroprevalence increased with age in 41% of the studies that examined this risk factor, as older people were more likely to have been exposed to arboviruses throughout their lifetime. Sex was also associated with seroprevalence in 13.5% of the studies; 14 of 185 studies reported that males exhibited higher seroprevalence than did females [15,16,20,28,78,82,92,96,115,123,126,133,135,138], whereas 11/185 studies reported higher significant seroprevalence among females [42,47,57,70,90,97,102,118,118,124,131]. Ethnicity was evaluated as a risk factor in seven studies conducted in Singapore (n = 4) [16,92,93,96,135], Colombia (n = 1) [54] and Laos (n = 1) [27]; these studies reported that the Indian, Afro-Colombian and Hmong–Mien ethnicities were likely to be seropositive. Although socio-economic status was measured in the studies using variable sets of markers, such as occupation, education, income, household size or access to drinking water, the main results indicated that persons of lower socio-economic status were more likely to be seropositive. Behavioral factors were evaluated in 11 of 185 studies, ten of which reported that protective behaviors (such as the use of vector control methods) were associated with being seronegative [55,64,70,71,71,76,90,97,126,164], supporting the hypothesis that the adoption of vector control measures will protect from infection; however, another study reported the opposite result [52]. Environmental factors, such as housing type and place, garbage collection or the presence of a potential mosquito breeding site, were associated with arboviruses seroprevalence in 11% of the studies [19,19,19,31,31,31,34,42,52,55,64,70,84,97,133,135,137,144,144,156]. Finally, 3% of the studies revealed an association between living in an urban area and being seropositive [26,26,57,75,89], which is not surprising because the main vector *A*. *aegypti* is an urban vector.

**Table 2:** Distribution of factors associated with arboviruses seroprevalence in overall studies and according to the arbovirus studied, N = 185.

Type of factors	Global	Dengue	Chikungunya	Zika
	N (%)	N (%)	N (%)	N (%)
**Age**				
*Older*	76 (41)	66 (44)	20 (37)	1 (10)
*Younger*	2 (1)	2 (1.3)	0	0
**Sex**				
*Women>Men*	11 (6)	7 (4)	4 (7.4)	0
*Men>Women*	14 (7.5)	9 (7)	6 (11)	0
**Ethnicity**	7 (4)	6 (4)	1 (1.8)	0
**Urban location**	5 (3)	5 (3.4)	0	0
**Environmental**	20 (11)	17 (11.4)	5 (9.2)	1 (10)
**Socio-economic**	19 (12)	16 (11)	4 (7.4)	0
**Behavioral**	11 (6)	9 (6)	1 (1.8)	1 (10)
TOTAL	185 (100)	149 (100)	54 (100)	10 (100)

#### Reporting of study biases

Sources of bias in the study design were identified by the authors in 76% of studies. Many different sources of bias were reported, most frequently the cross-reactivity among flaviviruses with ELISA tests for DENV and ZIKV. Sampling design was also frequently cited as lacking representativeness due to non-random sampling or small sample size. Other authors reported that they could not identify the serotypes through PRNT or distinguish between past and recent infection, that there was a recall bias (i.e., when a questionnaire was administered) or that the period was not optimal for the survey. For instance, some authors reported that they could not ensure that the observations of housing and environment made during the survey faithfully represented the conditions that prevailed at the time when seropositive persons were infected, possibly many years ago.

### Spatio-temporal distribution of seroprevalence studies worldwide

**Fig 3:**
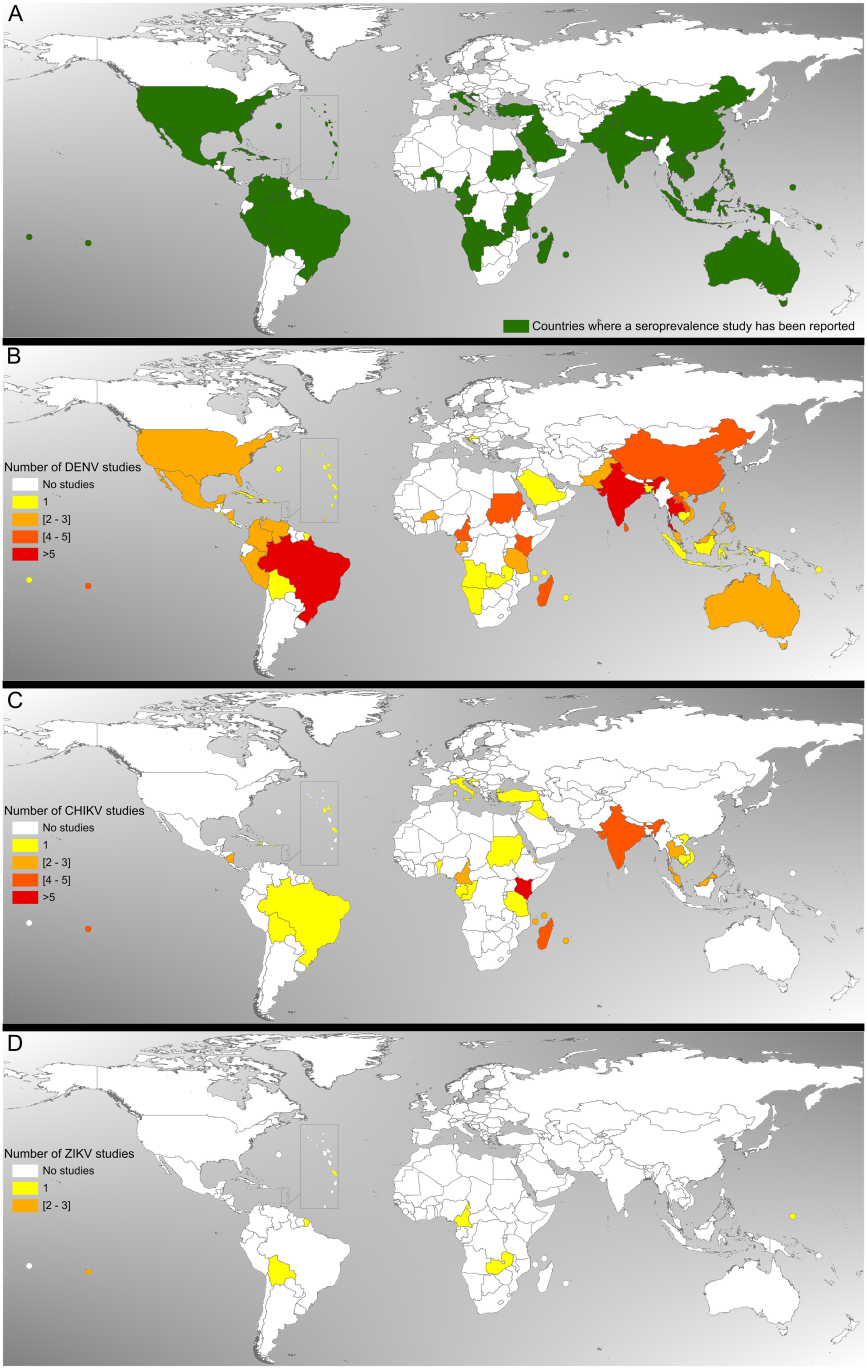
A) Distribution of arboviruses seroprevalence studies worldwide. B) Distribution of dengue seroprevalence studies number worldwide, 1989–2017, N = 149. C) Distribution of chikungunya seroprevalence studies number worldwide, 1989–2017, N = 54. D) Distribution of Zika seroprevalence studies number worldwide, 2007–2017, N = 10.

Overall, as shown in the maps in Fig 3A, the studies were primarily conducted in inter-tropical areas, with some disparities within this region. We identified data from eight world regions, including eight studies from North America [15,22,55,60,60,71], three from Europe [65,126,134], 12 from Oceania [8,29,32,33,114,161,163,165], 38 from Central America and the Caribbean [20,24,24,36–38,48,48,53,58,63,91, 94,95,97,98,100,103,105,106,111,117,117,118,120,121,121,141,158], 21 from Latin America [17,19,19,19,23,25,28,54,56,72,73,78–80,101,102,108,128,160,162], 44 from Africa [18,21,26,26,31,31,31,47,52,57,68,74,76,81,86,88,107,116,120,120,124,127,130–132,132,133,137,140,143,144,144–149,149,149,149,151,153,156,158], and 59 from Asia [16,27,30,34,35,39,41–45,45,46,49–51,59,61,62,62,62,62,64,66,67,69,70,75,77,82–85,87,89,89,90,92,93,96,99,104,109,110,112,113,115,117,123,125,129,135,136,138,139,141,150,152,152].

Dengue studies were primarily conducted in the Americas (39%) and in Asia (33%) (Fig 3B). The countries most heavily involved in the implementation of the surveys over the past two decades were Brazil (12 studies) [17,19,19,19,23,28,78–80,101,108,128], Singapore (ten studies) [16,35,62,62,62,62,82,92,93,96], Thailand (eight studies) [40,46,66,67,87,90,139,150] and India (seven studies) [34,45,45,69,75,138,152].

Chikungunya studies were primarily conducted in Africa (46%) and Asia (24%). The most represented countries were Kenya, with six studies, and India [123,125,138,152], Madagascar [128,128,128,128] and French Polynesia [114,163], with four studies (Fig 3C).

Finally, Zika studies were conducted in Oceania, the Caribbean, Africa and Latin America, with three studies in French Polynesia [161,165,165], one in Micronesia [8], one in the French Indies (Martinique) [159], one in Zambia [156], one in Cameroon [158], one in French Guiana [160] and one in Bolivia [162] (Fig 3D).

**Fig 4:**
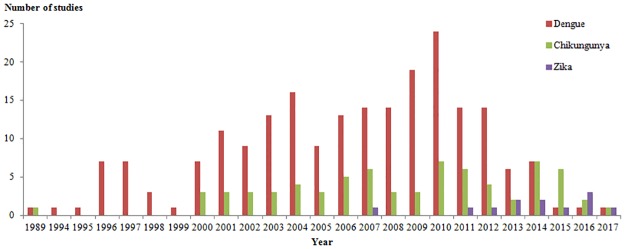
Distribution of dengue, chikungunya and Zika studies according to the year of survey (1989–2017; N = 185). If a survey was conducted over several years, we plotted this study for each given year.

The inclusion criteria restricted the analysis to studies published between January 2000 and March 2018; however, 14 studies were conducted before 2000 (Fig 4).

DENV seroprevalence studies were conducted between 1989 and 2017. Their distribution over the last decade indicated two peaks, one in 2004 and one in 2009–2010. The number of studies observed in 2004 might be enhanced by the re-emergence of CHIKV in Africa and the large DENV epidemic in Reunion Island. In 2010, the first phase III clinical trial for the now available tetravalent vaccine was initiated. This event may have encouraged seroprevalence studies to provide data for future vaccine programs. There were difficulties in interpreting the distribution of DENV studies with respect to the study year and location given the expansion of the virus in the Pacific, Southeast Asia, Africa, the Americas and the Middle East before the 1990s [166]. Moreover, at the time of this study, many countries were hyper-endemic with the co-circulation of four serotypes and with repeated epidemics every three to five years.

The first exclusive CHIKV seroprevalence study was conducted in 2004 in Kenya, with the re-emergence of the virus causing a large outbreak in 2004 [130]. This study was followed in 2005 by two studies, one in the Grande Comoros Island [131], where an outbreak occurred, and in Mayotte before the 2006 epidemic [132]. In 2006, four studies were conducted on Reunion Island and Mayotte during and after the 2006 epidemic [120,120,132,133] and in Benin, where no cases have been reported [116]. In 2007, three studies were conducted: one in Malaysia [129] (after the 2006 outbreak) when the virus subsequently spread to Asia, one in Gabon before the 2007 outbreak [153] and one in Italy [126] when CHIKV was imported to Europe, causing an outbreak. In 2008, two studies were conducted in India and Malaysia, where two outbreaks occurred [115,125]. In 2009, two studies were conducted in India and Kenya [123,124], and in 2010, a study was conducted in Congo after a 2010 CHIKV outbreak [127]. In 2013, CHIKV emerged in the Americas, and in 2014 and 2015, five studies were conducted in the Caribbean [118,118,119,121] (Saint-Martin, Guadeloupe, Martinique and Puerto Rico) and Central America [122] (Nicaragua) during an outbreak in Saint Martin and post-outbreak in the other locations. One study was conducted in Vietnam in 2015, where little was known about CHIKV transmission and where dengue is endemic [136]. The last study was conducted in 2016 in Brazil in a post-outbreak context [128].

There were seven ZIKV studies. The first study was conducted in Yap Island during the 2007 outbreak [8], and the second study was conducted in Zambia in 2013 [156], where no information on ZIKV was available. Two studies were conducted in French Polynesia in two distinct populations during and after the 2014 outbreak and one in 2015 [155]. Another study was conducted in Cameroon in 2015 [158], and one study was conducted in 2016 in Martinique (West Indies) [159]. Finally, the last study was conducted in French Guiana during the ZIKV outbreak in 2016 [160].

#### Seroprevalence studies of multiple viruses

We observed that DENV and CHIKV studies were primarily conducted during and after the year 2004, when CHIKV re-emerged in Kenya and spread rapidly though new areas. Two studies were conducted in Kenya [144,144] and one in Cameroon [147] before the CHIKV outbreak in 2004, but these studies focused on several arboviruses affecting those two countries, including CHIKV, yellow fever virus (YFV), tick-borne encephalitis virus, DENV, sindbis virus, o’nyong nyong virus and Tahyna virus.

Three studies, one on DENV and ZIKV [161] and two on DENV and CHIKV [167,167], were conducted in French Polynesia, a territory already hyper-endemic for DENV. The first study was conducted in 2013, immediately before the emergence of ZIKV, whereas the two additional studies were conducted after the emergence of ZIKV and CHIKV in 2014.

One study on both viruses was conducted in Bolivia in 2017, where few seroprevalence results in the region have been made available [162].

### Arboviruses seroprevalence

All seroprevalence data are presented in S1 Appendix.

#### Dengue seroprevalence studies

**Fig 5:**
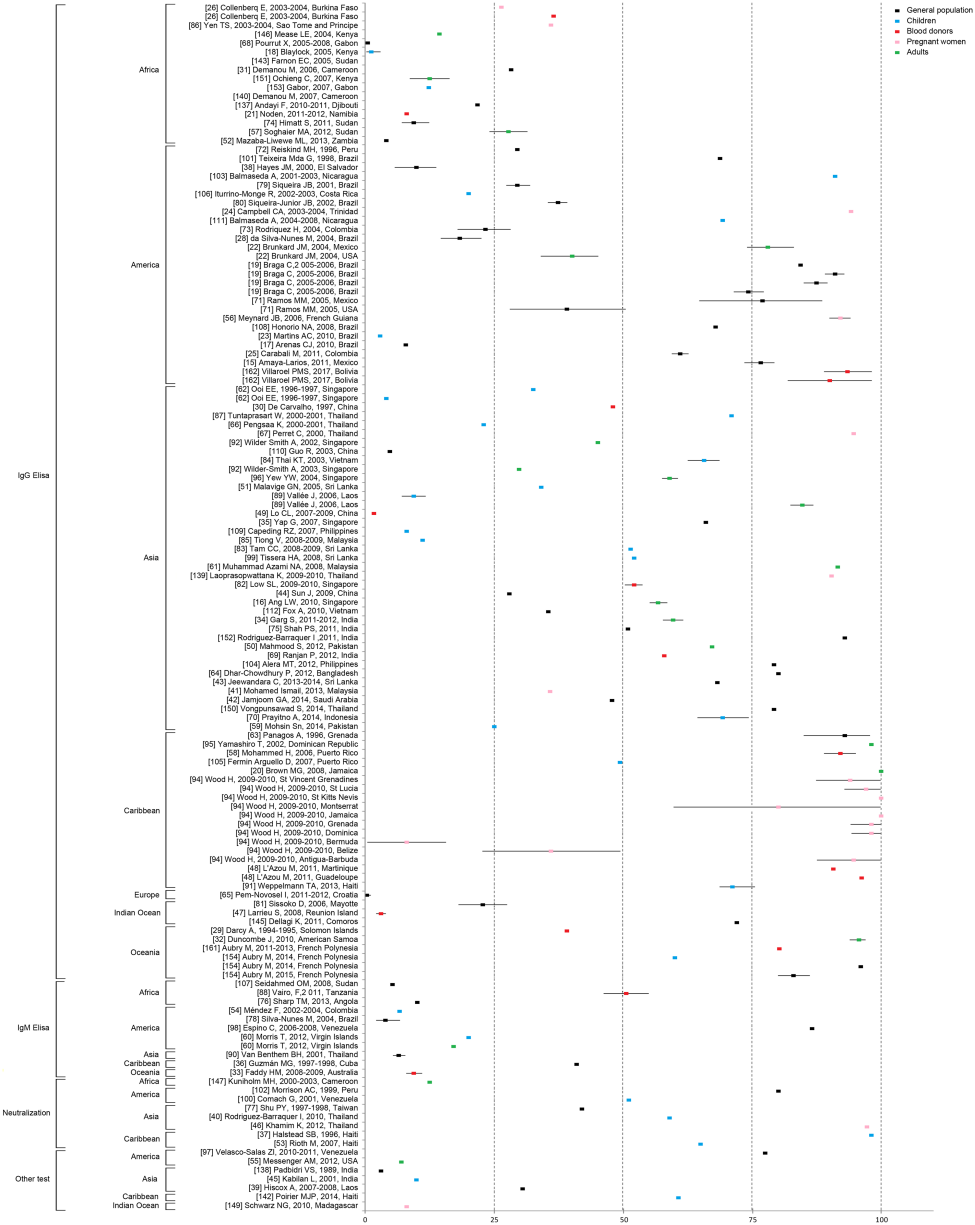
Dengue seroprevalence rates by assay and continent, according to subpopulations. Each square and associated 95% confidence interval were derived from an individual study (see S1 Appendix). If a study was conducted in different places within a given country, only the overall mean seroprevalence was included in this figure.

Seroprevalence data are presented in Fig 5. The seroprevalence of DENV ranged from less than 1% [62,65,68,140,143,168] to 100% in a study conducted among 442 pregnant women in the Caribbean Islands (St Kitts Nevis and Jamaica) [94]. Among studies that performed IgG ELISA, the highest seroprevalence rates were observed in the Caribbean region and in the Americas. Studies conducted in Asia highlighted lower seroprevalence rates, ranging from 50 to 75%. Seroprevalence rates appeared to be the lowest in Africa, ranging from 0 to 35%.

Overall, analysis revealed that DENV seroprevalence in the Americas was higher than that in Asia (64.4% vs. 46.2%, p<0.01), which was higher than that in Africa (46.2 vs. 18.1%, p<0.001). Seroprevalence data were not available for one study conducted in Tanzania, where multiple arboviruses co-circulate; in the corresponding analysis, positivity for at least one DENV serotype, or West Nile and/or yellow fever virus antibodies, was categorized as positive for “flavivirus IgG” [148].

We observed that in the Americas, DENV seroprevalence exhibited substantial variations, differing by as much as 50 percentage points between states. Moreover, within a country, the seroprevalence could also vary considerably; for instance, in Brazil, the seroprevalence ranged from 3% to 90%.

#### Chikungunya seroprevalence studies

**Fig 6:**
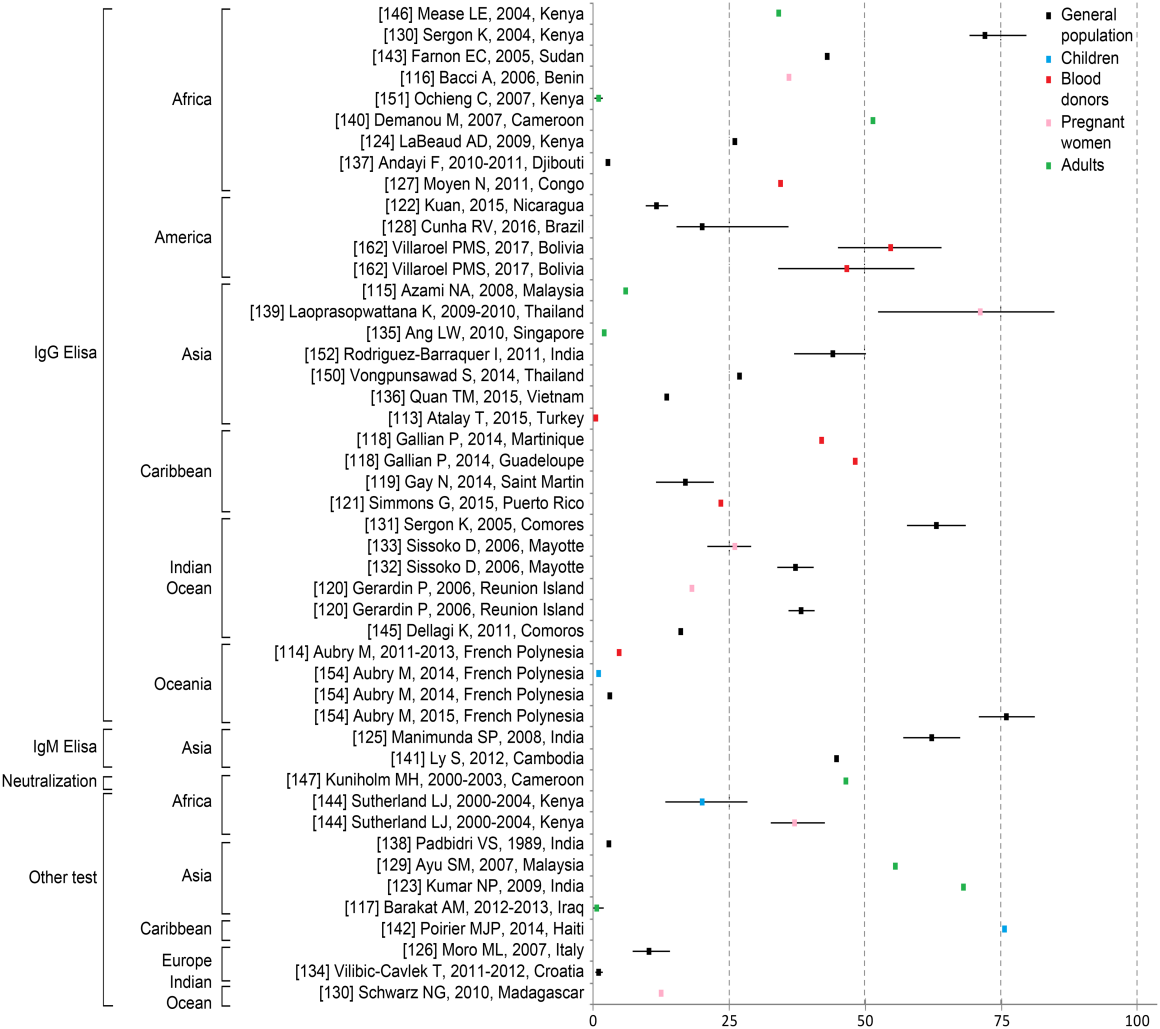
Chikungunya seroprevalence rates by assay and continent, according to subpopulations. Each square and associated 95% confidence interval were derived from an individual study (see S1 Appendix). If a study was conducted in different places within a given country, only the overall mean seroprevalence was included in this figure.

Seroprevalence data are presented in Fig 6. The seroprevalence of CHIKV ranged from 0.4% in a study sample of 500 blood donors from an urban area in Central Anatolia in Turkey [113] to 75.6% in a study conducted among 127 children living in an urban area in Haiti [142] and 76.0% in a study conducted among residents in French Polynesia [163]. Although only a small number of countries were represented worldwide, we noted that CHIKV seroprevalence was the highest among the general population of Lamu Island (Kenya) at 72% (95% confidence interval (CI): 69–79) [130]; among pregnant women in Thailand at 71.2% (95% CI: 52–84) [139] and among the general population of French Polynesia at 76% (95% CI: 71–81) [163], with both studies performing IgG ELISA; and among Haitian children at 75.6% (performing multiplex assay) [142] Variations within the same country were also observed in Kenya, where CHIKV seroprevalence varied substantially, between 1% and 72% [131,151].

#### Zika seroprevalence studies

**Fig 7:**
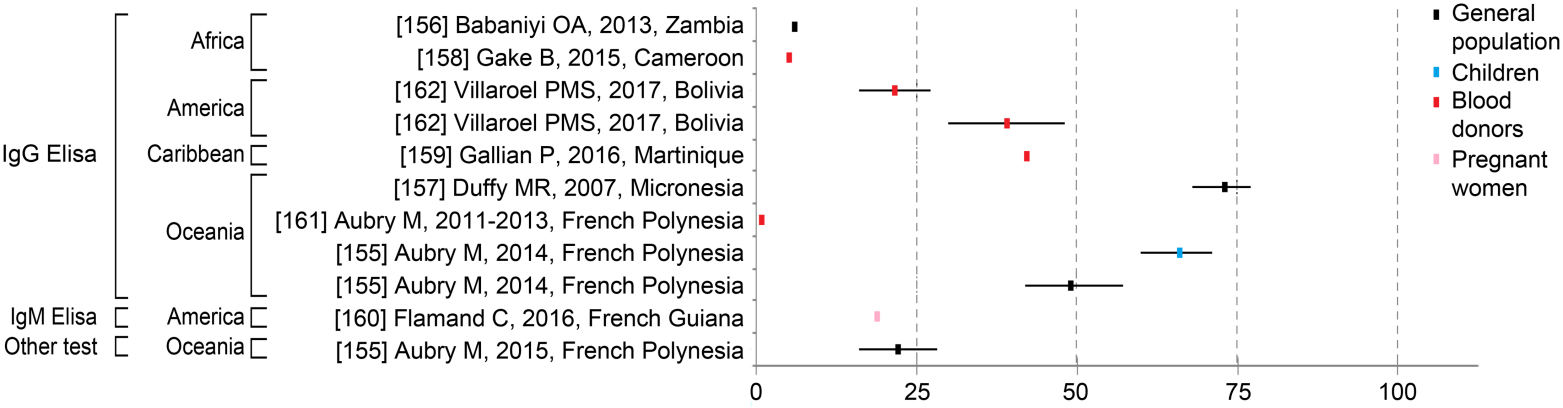
Zika seroprevalence rates by assay and continent, according to subpopulations. Each square and associated 95% confidence interval were derived from an individual study (see S1 Appendix). If a study was conducted in different places within a given country, only the overall mean seroprevalence was included in this figure.

ZIKV seroprevalence data were drawn from ten studies worldwide in which IgG and IgM ELISA diagnostic tests were performed, as were microsphere immunoassays in one study. The seroprevalence was the highest in Yap Island, Micronesia, at 73% (95% CI: 68–77) [8] and in French Polynesia at 66% (95% CI: 60–71) [155]. In French Polynesia, three studies reported seroprevalence ranging from less than 1% among blood donors to more than 50% among children, as they were conducted before and after the emergence of ZIKV, respectively (Fig 7) [155,161]. We noted that in the Americas, ZIKV seroprevalence rates ranged between 19 and 30% [160,162], and in Africa, rates were less than 10% [156,158].

#### Seroprevalence stratified by age

Seroprevalence was stratified by age in 72 studies primarily conducted among the general population (53%, p<0.001). The data were sufficient to describe the seroprevalence among children/adolescents (< 18 years) and adults (≥18 years). When studies presented data for 14- to 20-year-olds, members of this group were categorized as children/adolescents. Among CHIKV studies, there were no differences in seroprevalence between adults and children. This finding may be explained by the recent introduction of the virus into countries involved in each study. Overall, with respect to DENV infection, seroprevalence was higher among adults (58.1%; SD: 4.5) than among children (38.4%; SD: 4.1; p<0.01).

## Discussion

Arboviral infections are common causes of disabling fever syndromes worldwide. In many countries, the concomitant co-circulation of dengue, chikungunya and Zika viruses represents a major recent public health and biomedical challenge. Prior to the introduction and subsequent spread of CHIKV and ZIKV in the Americas, dengue was the predominant arboviral infection worldwide. In this context of emerging and re-emerging arboviral diseases worldwide, estimating the burden of these diseases represents a major challenge to more efficient planning for disease control and reducing the risk of future re-emergence of arboviruses. Several affected countries face challenges in estimating the burden of arboviruses. Nonetheless, estimating the true burden of arboviral infections remains a difficult task given the large number of unapparent infections, especially those of dengue fever [1]. Serological surveys are thus required to identify the distribution of these diseases and measure their epidemic impact. A recent estimate indicated that the number of cases affected by any of these three arboviruses dramatically increased after 2013, reaching over 3.5 million by the end of 2015 in the Americas [169].

This review emphasizes several aspects of arboviruses epidemiology and describes current challenges and implications for dengue, chikungunya and Zika seroprevalence studies worldwide.

Overall, our results highlight the highly heterogeneous nature of study designs and serological tests used in arboviral seroprevalence studies. Seroprevalence surveys have the benefit of not being affected by surveillance system sensitivity or symptomatic case reporting rates but still have several limitations inherent to the adopted methodology. Selection biases, defined in our review as a distortion in the seroprevalence rate, may occur due to the use of a non-probabilistic sampling frame or poor field worker practices, such as replacing a selected household with one that is easier to reach [170]. Furthermore, the use of serum samples collected for various purposes frequently hinders the representativeness of the population sample and, consequently, that of the provided estimations. Even if the use of convenience samples is a good strategy for increasing the volume of serological data produced, the potential biases such sampling introduces must be considering during the analysis process to produce valid results. These limitations in the literature underscore the challenge of estimating global prevalence in the absence of nationally representative age-specific databases. Whenever surveys are conducted, all efforts to ensure high-quality collected data should be made. In particular, probabilistic samples should be used, and the sample size and number of clusters should be selected appropriately to rigorously estimate population seroprevalence rates.

The review also highlights the variety of serological tests used to measure antibodies activities. ELISA tests are the most common diagnostic method (used in more than half of the studies included in this review). Moreover, we noted that more than half of the studies that performed IgG ELISA tests used the indirect method, which is recommended, as it allows for the detecting of lower levels of antibodies than the direct method does and is thus more sensitive [171]. Most commercially available diagnostic IgG ELISAs that are adjusted to measure past arboviral exposure tend to have high sensitivity but suffer from low specificity due to high cross-reactivity with other arboviruses (flaviviruses or alphaviruses) circulating in a given geographical area or with Japanese encephalitis (JE) and YFV recommended immunization [172,173]. The resulting false positives could lead to information bias that can be overcome through control with neutralization tests. Both tests provide complementary results because one test is a biochemical assay (ELISA) measuring antibodies binding to the antigen and the other is a biological assay measuring antibodies’ capacity to neutralize an infecting virus. Only neutralization tests measure the biological parameters of *in vitro* virus neutralization and are the most virus-specific serological tests [174]. Indeed, for seroepidemiological studies, neutralization tests remain the “gold standard” for confirming and serotyping DENV infections in regions where two or more flaviviruses are co-circulating. These tests, however, are time-consuming, labor-intensive and expensive and are not as amenable to testing large numbers of sera as the ELISA is. When neutralization tests are not performed to complete results from IgG ELISA, country-specific contexts, including the presence of other circulating flaviviruses or alphaviruses and immunization programs for JE and YF, must be considered when interpreting seroprevalence results.

Although we stratified seroprevalence data by assay to allow for comparisons, more than half of the ELISA tests were performed using different commercial kits and in-house assays with variable sensitivities and specificities. Moreover, differences in assay formats, usage of antigen, and detection systems make it difficult to estimate the performances value of each individual assay by proper comparison [175]. In a multicenter evaluation using a commercial assay, it was shown that the sensitivities and the specificities varied between studies depending on the serum samples of the respective collaborating centers used for the performance evaluation [176]. These variations reported from several studies [177] indicated the need to develop the most sensitive and specific diagnosis tool to provide recommendations for future serological studies.

Although the diversity of study designs and serological tests used in the selected seroprevalence studies represents a major limitation for the comparison of seroprevalence rates by geographical region, our literature search highlights the highly heterogeneous seroprevalence of DENV and CHIKV worldwide as well as the significant variability among regions in the same country. The review also clearly shows that seroprevalence was the highest in island environments for both arboviruses. Some of these variations may stem from methodological differences, as well as the choice of study population, sample size and diagnostic test. This heterogeneity may also reflect differential exposure to mosquitoes. Indeed, disease transmission can substantially differ between regions characterized by different environmental and climatic determinants of vector density. For instance, in Kenya, alphavirus antibodies, specifically those against CHIKV, were detected only in children from the Kisumu District (lowlands) and not in children from the Nandi District (highlands) [144]. Geographic and climatic differences between these two regions could provide evidence for varying environmental factors related to arboviruses transmission risk. For instance, mosquito vectors are not as prolific in the colder climate of the highlands, whereas the lowlands offer warmer and wetter areas for mosquito development and could provide an appropriate environment for mosquito vectors and subsequent arboviral transmission. Moreover, the seroprevalence heterogeneity is related to the different transmission dynamics of these arboviruses, including force of infection, reproductive number and others factors such as strain [178].

The review highlights some factors associated with arboviral seroprevalence. We noted an increase in seroprevalence among older people in 44% of DENV studies and 18.5% of CHIKV studies mainly conducted in endemic areas, whereas only one ZIKV study obtained this result. The epidemiological context of affected countries appears to be associated with the relationship between age and seroprevalence. For instance, in an emergence context, few studies reported this association, as the target population is naïve. However, some CHIKV studies not conducted in an endemic area reported this association, which may suggest that age is associated with level of exposure. We noted that 14 studies found an association between gender and seroprevalence. These findings suggest that there may be gender-related differences; however, these discrepancies require further exploration, as the health gender gap may stem from various patterns that affect exposure to mosquitoes [179]. We also observed that, regarding DENV and CHIKV, this association varied between countries, and no study reported an association between gender and ZIKV seroprevalence. However, given the transmission issues associated with ZIKV, we can expect that in future studies, sexual transmission will correlate with higher seroprevalence among women. Although few studies revealed an association between ethnicity and seroprevalence, this finding can be related to background prevalence in the country of origin, in combination with increased early life exposure before migration or exposure during travel to their region of origin post migration. Moreover, these associations might also be partly explained by increased susceptibility related to the lower socioeconomic position of certain ethnic groups. The relationship between health and socio-economic status is well documented, and research has revealed a graded association in which people of lower socio-economic status have much worse health outcomes than those of higher socio-economic status [180,181]. Finally, 20 studies also reported an association between environmental factors and seroprevalence, as certain environmental conditions, such as house structure or objects collected in the yard, are more hospitable to *A*. *aegypti* [182].

Although the proportion of seropositivity depends on the diagnostic method used, it also relies on study planning; if a serosurvey is conducted long after the end of an outbreak, the signal for the antibodies may be lower than in a study conducted close to the end of an outbreak. Our results indicate that DENV seroprevalence in the Americas was higher than that in Asia, which is surprising because dengue has been endemic in Southeast Asia for decades [183], and the Asian burden, including the Western Pacific, accounted for nearly 75% of the global burden worldwide [184]. Analysis revealed that studies conducted in the Americas were performed significantly more frequently in an outbreak or post-outbreak context (p<0.01) via IgM ELISA, which could explain the discrepancy between America and Asia.

Our review included 185 studies worldwide according to well-defined inclusion criteria. The findings indicate that the distribution of our studies follows the same pattern observed for the expansion of their vectors [185]. Moreover, all of the studies reflect areas of arboviral circulation in an epidemic pattern. However, the maps clearly demonstrated where seroprevalence survey data are lacking and identified potential places for implementing future seroprevalence studies; the maps also highlighted places in tropical regions where no data are available, especially for CHIKV and ZIKV, which are considered emergent or re-emergent viruses. Some countries located in the tropics and subtropics were not represented, although they are considered at risk of transmission by the WHO; this is especially true for Africa, where few studies were conducted and where the epidemiology of these arboviruses is under-exploited. In addition, a recent review suggested that dengue transmission is endemic to 34 countries in all regions of Africa [186].

The temporal distribution of Chikungunya studies followed the timeline of CHIKV outbreaks during its rapid expansion since 2004, suggesting that ZIKV surveys, in the context of its recent emergence in the Americas, may be currently in process and, if not, that such surveys should be addressed rapidly.

Serological surveys provide the most direct measurement for defining the immunity landscape for infectious diseases, but they remain difficult to implement. Overall, dengue, chikungunya and Zika serosurveys have followed the expansion of these arboviruses, but there remain gaps in their distribution. Serological studies can address future challenges in identifying trends in arboviruses transmission over time, and age-specific antibody prevalence rates can be used to estimate when major changes in transmission occurred.

## Supporting information

S1 Appendix Data characteristics extracted for each study included in the review. (PDF)

S1 Checklist PRISMA checklist. (PDF)
