# Supplementary material for: Current challenges and implications for dengue, chikungunya and Zika seroprevalence studies worldwide: A scoping review
Source: PLoS Negl Trop Dis. 2018 Jul 16;12(7):e0006533. doi: 10.1371/journal.pntd.0006533 (PMC6062120; doi:10.1371/journal.pntd.0006533)
Supplement: S1 Appendix — (PDF) [file pntd.0006533.s001.pdf]

Ref	Authors/ Year of publication	Country	Virus	Population type	Age-group	Recruitment	Year	Laboratory test	Sample size	Dengue*	Chikungunya*	Zika*
[107]	Alera MT et al/2016	Philippines	dengue	general population	all ages	household	2012	IgM+IgG ELISA&RT-PCR& HAI	1008	79.2		
[18]	Amaya-Larios IY et al/2014	Mexico	dengue	general population	all ages	household	2011	IgM+IgG ELISA & PRNT	929	76.6		
[140]	Andayi F et al /2014	Djibouti	dengue+chik	general population	all ages	household	2010-2011	IgG ELISA & Neutalization tests	1045	21.8	2.6	
[19]	Ang LW et al/2015	Singapore	dengue	age specific subgroup	adults	previous study	2010	NP	3293	56.8		
[138]	Ang LW et al/2017	Singapore	chik	age specific subgroup	adults	previous study	2010	IgG ELISA & PRNT	3293		1.9	
[20]	Arenas CJ et al/2012	Brazil	dengue	general population	all ages	household	2010	IgM +IgG ELISA	886	7.8		
[108]	Argüello DF et al/2010	Puerto Rico	dengue	age specific subgroup	children	school	2007	IgM+IgG ELISA & MNT	345	49.3		
[117]	Aubry M et al/2015	French polynesia	chik	blood donors	adults	hospital	2011-2013	IgG ELISA	593		4.7	
[163]	Aubry M et al/2015	French polynesia	dengue+zika	blood donors	adults	hospital	2011-2013	IgG ELISA	593	80.3		0.8
[157]	Aubry M et al/2018	French polynesia	dengue+chik	general population	all ages	household	2014	IgG ELISA	196	96	3	
[157]	Aubry M et al/2018	French polynesia	dengue+chik	schoolchildren	children/ado	school	2014	IgG ELISA	476	60	1	
[157]	Aubry M et al/2018	French polynesia	dengue+chik	general population	all ages	household	2015	MIA	700	83	76	
[158]	Aubry M et al/2017	French polynesia	zika	schoolchildren	children/ado	school	2014	IgG ELISA	476			66
[158]	Aubry M et al/2017	French polynesia	zika	general population	all ages	household	2014	IgG ELISA& MIA	196			49
[158]	Aubry M et al/2017	French polynesia	zika	general population	all ages	household	2015	MIA	700			22
[132]	Ayu SM et al/2010	Malaysia	chik	age specific subgroup	ado/adult	household	2007	Neutralization Test	180		55.6	
[118]	Azami NA et al/2013	Malaysia	chik	age specific subgroup	adults	previous study	2008	IgG ELISA	945		5.93	
[166]	Babaniyi OA et al/2015	Zambia	zika	general population	all ages	household	2013	IgM +IgG ELISA	3625			6
[119]	Bacci A et al/2015	Benin	chik	pregnant women	ado/adult	hospital	2006	IgM+IgG ELISA & IIFT & MNT	352		36	
[106]	Balmaseda A et al/2006	Nicaragua	dengue	schoolchildren	children/ado	school	2001-2003	IgM+IgG ELISA&RT-PCR & PRNT & HI	999	91		
[114]	Balmaseda A et al/2010	Nicaragua	dengue	children	children	household	2004-2008	IgM+IgG ELISA & RT-PCR& IIFT	3800	69.3		
[120]	Barakat AM et al/2016	Iraq	chik	age specific subgroup	adults	center recruitment	2012-2013	HI & IFA & MNT	200		0.5	
[21]	Blaylock JM et al/2011	Kenya	dengue	age specific subgroup	infant/children	previous study	2005	IgG ELISA	354	1.1		
[22]	Braga C et al/2010	Brazil	dengue	general population	all ages	household	2005-2006	IgG ELISA	976	91.1		
[22]	Braga C et al/2010	Brazil	dengue	general population	all ages	household	2005-2006	IgG ELISA	923	87.4		
[22]	Braga C et al/2010	Brazil	dengue	general population	all ages	household	2005-2006	IgG ELISA	920	74.3		

[23]	Brown MG et al/2009	Jamaica	dengue	NP	NP	hospital		IgM +IgG ELISA	277	100		
[25]	Brunkard JM et al/2007	USA	dengue	age specific subgroup	adults	household	2004	IgM+IgG ELISA	300	78		
[25]	Brunkard JM et al/2007	Mexico	dengue	age specific subgroup	adults	household	2004	IgM+IgG ELISA	300	40		
[27]	Campbell CA et al/2007	Trinidad	dengue	pregnant women	NP	hospital	2003-2004	IgM +IgG ELISA	79	95		
[27]	Campbell CA et al/2007	Trinidad	dengue	pregnant women	NP	hospital	2003-2004	IgM +IgG ELISA	46	93.5		
[112]	Capeding RZ et al/2010	Philippines	dengue	age specific subgroup	infant	hospital	2007	IgM+IgG ELISA	250	8		
[28]	Carabali et al/2017	Colombia	dengue	general population	all ages	household	2011	IgG ELISA & PRNT	3684	61		
[29]	Collenberg E et al/2006	Burkina Faso	dengue	pregnant women	adults	hospital	2003-2004	IgG ELISA	289	26.3		
[29]	Collenberg E et al/2006	Burkina Faso	dengue	blood donors	adults	hospital	2003-2004	IgG ELISA	102	36.5		
[103]	Comach G	Venezuela	dengue	age specific subgroup	children/ ado	NP	2001-2002	PRNT	710	51		
[30]	Conlan JV et al/2015	Laos	dengue	general population	all ages	household	2009	HI	1419	***		
[131]	Cunha RV et al/2017	Brazil	chik	general population	all ages	household	2016	IgM +IgG ELISA	120		20	
[31]	da Silva-Nunes M et al/2008	Brazil	dengue	general population	all ages	household	2004	IgM+IgG ELISA & PRNT & RT-PCR	405	18.3		
[32]	Darcy A et al/2001	Solomon Islands	dengue	blood donors	adults	hospital	1994-1995	IgG ELISA & PRNT	515	39		
[33]	De Carvalho IL et al/2011	China	dengue	blood donors	adults	blood donors center	1997	Western Blot	753	48		
[148]	Dellagi K et al/2016	Comoros	dengue+chik	general population	all ages	laboratory	2011	IgM+IgG ELISA & MNT	75	72	16	
[34]	Demanou M et al 2014	Cameroon	dengue	general population	all ages	household	2006	IgM+IgG ELISA & PRNT	728	24.2		
[143]	Demanou M et al/2010	Cameroon	dengue+chik	age specific subgroup		center recruitment	2007	IgG ELISA	105	0	51.4	
[34]	Demanou M et al/2014	Cameroon	dengue	general population	all ages	household	2006	IgM+IgG ELISA & PRNT	699	61.4		
[34]	Demanou M et al/2014	Cameroon	dengue	general population	all ages	household	2007	IgM+IgG ELISA & PRNT	603	9.8		
[67]	Dhar-Chowdhury P et al/2017	Bangladesh	dengue	general population	all ages	household	2012	IgM+IgG ELISA & PRNT	1125	80		
[10]	Duffy MR et al/2009	Micronesia	zika	general population	all ages	household	2007	IgM ELISA	557			73
[35]	Duncombe J et al/2013	American Samoa	dengue	age specific subgroup	adults	household	2010	IgG ELISA	807	95.6		
[100]	Espino C et al/2010	Venezuela	dengue	general population	all ages	household	2006-2008	IgM ELISa & PRNT& RT-PCR	2125	86.6		
[36]	Faddy HM et al/2013	Australia	dengue	blood donors	adults	blood donors center	2008-2009	IgM ELISA	3037	0.13		

[36]	Faddy HM et al/2013	Australia	dengue	blood donors	adults	blood donors center	2008-2009	IgM ELISA	2416	0.33		
[146]	Farnon EC et al/2010	Sudan	dengue+chik	general population	all ages	household	2005	IgM+IgG ELISA & PRNT	87	0	43	
[162]	Flamand C et al/2017	French Guiana	zika	Pregnant women	ado/adult	laboratory	2016	IgM ELISA & PCR	3050			18.8
[115]	Fox A et al/2014	Vietnam	dengue	general population	all ages	household	2010	IgG ELISA & PRNT	606	35.5		
[156]	Gabor JJ et al/2016	Gabon	dengue+chik	age specific subgroup	infants		2007 -10	IgG ELISA	162	12.3	0.6	
[160]	Gake B et al/2017	Cameroon	zika	blood donors	adults	NP	2015	IgG ELISA & MNT	1084			5
[161]	Gallian P et al/2017	Martinique	zika	blood donors	adults	blood donors center	2016	IgG ELISA & MNT	176			42.2
[121]	Gallian P et al/2017	Martinique	chik	blood donors	adults	blood donors center	2014	IgG ELISA & RTPCR	5669		41.9	
[121]	Gallian P et al/2017	Guadeloupe	chik	blood donors	adults	blood donors center	2014	IgG ELISA & RTPCR	2984		48.1	
[37]	Garg S et al/2016	India	dengue	age specific subgroup	children	household	2011-2012	IgG ELISA & PRNT	2591	59.6		
[122]	Gay N et al/2016	Saint Martin	chik	general population	all ages	laboratory	2014	IgM +IgG ELISA	203		16.9	
[123]	Gérardin P et al/2008	Reunion Island	chik	pregnant women	NP	laboratory	2006	IgM+IgG ELISA	888		18.2	
[123]	Gérardin P et al/2008	Reunion Island	chik	general population	all ages	household	2006	IgM+IgG ELISA	2242		38.2	
[113]	Guo R et al/2014	China	dengue	general population	all ages	hospital	2003	IgG ELISA	187	4.81		
[109]	Iturrino-Monge R et al/2006	Costa Rica	dengue	age specific subgroup	children	hospital	2002-2003	IgG ELISA	206	20		
[125]	Kuan et al/2016	Nicaragua	chik	age specific subgroup	children/ado	previous study	2015	IgG ELISA	3362		6.1	
[125]	Kuan et al/2016	Nicaragua	chik	age specific subgroup	adults	household	2015	IgG ELISA	848		13.1	
[39]	Guzmán MG et al/2000	Cuba	dengue	general population	all ages	household	1997-1998	IgM ELISA & neutralization tests	1151	41		
[40]	Halstead SB et al/2001	Haiti	dengue	schoolchildren	children/ado	school	1996	PRNT & Sequencing	210	98		
[41]	Hayes JM et al/2003	El Salvador	dengue	general population	all ages	household	2000	IgM+IgG ELISA	373	9.8		
[77]	Himatt S et al/2015	Sudan	dengue	general population	all ages	household	2011	IgM+IgG ELISA	491	9.4		
[42]	Hiscox A et al/2010	Laos	dengue	general population	all ages	household	2007-2008	HI	1708	30.4		
[111]	Honorio NA et al/2009	Brazil	dengue	general population	all ages	household	2008	IgM+IgG ELISA & RT-PCR	258	67.8		
[45]	Jamjoom GA et al/2016	Saudi Arabia	dengue	general population	all ages	hospital	NP	IgG ELISA	1939	47.8		
[46]	Jeewandara C et al /2015	Sri Lanka	dengue	general population	all ages	hospital	2013-2014	IgG ELISA	1689	68.2		
[48]	Kabilan L et al/2004	India	dengue	schoolchildren	children/ado	school	2001	HI	229	2.2		
[48]	Kabilan L et al/2004	India	dengue	schoolchildren	children/ado	school	2001	HI	209	9.9		
[49]	Khamim K et	Thailand	dengue	pregnant women	ado/adult	hospital	2012	PRNT	141	97.2		

	al/2015										
[126]	Kumar NP et al/2011	India	chik	age specific subgroup	adults	household	2009	IFA	381		68
[150]	Kuniholm MH et al/2006	Cameroon	dengue+chik	age specific subgroup	adults	previous study	2000-2003	PRNT	256		46.5
[127]	LaBeaud AD et al/2015	Kenya	chik	general population	all ages	previous study	2009	IgG ELISA & PRNT	1848		26
[142]	Laoprasopwattana K et al/2016	Thailand	dengue+chik	pregnant women		hospital	2009-2010	IgM+IgG ELISA	319	90.3	71.2
[50]	Larrieu S et al/2014	Reunion Island	dengue	blood donors	adults	blood donors center	2008	IgG ELISA	1825	3.1	
[51]	L'Azou M et al/2015	Guadeloupe	dengue	blood donors	adults	blood donors center	2011	IgG ELISA & Neutralization test	397	96.2	
[51]	L'Azou M et al/2015	Martinique	dengue	blood donors	adults	blood donors center	2011	IgG ELISA & Neutralization test	386	90.7	
[52]	Lo CL et al/2013	China	dengue	blood donors	adults	blood donors center	2007-2009	IgG ELISA	685	1.6	
[85]	Low SL et al/2015	Singapore	dengue	blood donors	adults	blood donors center	2009-2010	IgM+IgG ELISA & PRNT	3995	52	
[54]	Malavige GN et al/2006	Sri Lanka	dengue	schoolchildren	children/ado	school		IgG ELISA & HI	313	34.1	
[128]	Manimunda SP et al/2010	India	chik	general population	all ages	household	2008	IgM ELISA	360		62.2
[26]	Martins AC et al/2014	Brazil	dengue	age specific subgroup	infant/children	household	2010	IgG ELISA	411	2.9	
[55]	Mazaba-Liwewe ML et al/2014	Zambia	dengue	general population	all ages	household	2013	IgG ELISA	3624	4.1	
[149]	Mease LE et al/2011	Kenya	dengue+chik	age specific subgroup	adults	center recruitment	2004	IgG ELISA	1141	14.4	34
[57]	Mendez F et al/2006	Colombia	dengue	schoolchildren	children/ado	school	2002-2004	IgM ELISA & IFA	3189	6.7	
[58]	Messenger AM et al/2014	USA	dengue	age specific subgroup	adults	center recruitment	2012	IgM+IgG RDT**	173	6.9	
[59]	Meynard JB et al/2009	French Guiana	dengue	pregnant women	ado/adult	hospital	2006	IgM +IgG ELISA	689	92	
[44]	Mohamed Ismail NA et al/2014	Malaysia	dengue	pregnant women	NP	hospital	NP	IgM +IgG ELISA	358	35.8	
[61]	Mohammed H et al/2012	Puerto Rico	dengue	blood donors	adults	blood donors center	2006	IgG ELISA & MNT	300	92	
[62]	Mohsin SN et al/2016	Pakistan	dengue	NP	infant/children	household	NP	Ig G ELISA	400	25	
[129]	Moro ML et al/2010	Italy	chik	general population	all ages	household	2007	IFA	325		10.2
[63]	Morris T et al/2013	Virgin Islands	dengue	schoolchildren		school	2012	IgM ELISA & PCR	203	20	

[63]	Morris T et al/2013	Virgin Islands	dengue	age specific subgroup	adults	center recruitment	2012	IgM ELISA & PCR	118	17	
[105]	Morrison AC et al/2010	Peru	dengue	general population	all ages	household	1999	PRNT	2400	80	
[130]	Moyen N et al/2014	Congo	chik	blood donors	adults	blood donors center	2011	IgG ELISA & Sequencing	517		34.4
[64]	Muhammad Azami NA et al/2011	Malaysia	dengue	age specific subgroup	adults	previous study	2008	IgG ELISA	1000	91.6	
[24]	Noden BH et al/2014	Namibia	dengue	blood donors	adults	blood donors center	2011-2012	IgG ELISA	312	8	
[154]	Ochieng C et al/2015	Kenya	dengue+chik	age specific subgroup	adults	household	2007	IgG ELISA	1091	12.5	0.97
[65]	Ooi EE et al/2001	Singapore	dengue	age specific subgroup	children/ado	hospital	1996-1997	IgG ELISA	72	6.5	
[65]	Ooi EE et al/2001	Singapore	dengue	age specific subgroup	infant	hospital	1996-1997	IgG ELISA	126	32.5	
[65]	Ooi EE et al/2001	Singapore	dengue	age specific subgroup	children	hospital	1996-1997	IgG ELISA	389	0.77	
[65]	Ooi EE et al/2001	Singapore	dengue	age specific subgroup	children	hospital	1996-1997	IgG ELISA	297	6.7	
[141]	Padbidri VS et al/2002	India	dengue+chik	general population	all ages	household	1989	HI	2401		2.9
[66]	Panagos A et al/2005	Grenada	dengue	general population	all ages	household	1996	IgG ELISA	75	93	
[68]	Pem-Novosel I et al/2015	Croatia	dengue	general population	all ages	hospital	2011-2012	IgG ELISA & IFA	1180	0.6	
[69]	Pengsaa K et al/2008	Thailand	dengue	age specific subgroup	infant/children	hospital	2000-2001	IgM+IgG ELISA & PRNT& HI	447	23	
[70]	Perret C et al/2005	Thailand	dengue	pregnant women	ado/adult	hospital	2000	IgM+IgG ELISA & HAI	245	94.7	
[145]	Poirier MJP et al/2016	Haiti	dengue+chik	age specific subgroup	children/ado	household	2014	Luminex	127	60.6	75.6
[71]	Pourrut X et al/2011	Gabon	dengue	general population		household	2005-2008	IgM+IgG ELISA	4341	0.5	
[73]	Prayitno A et al/2017	Indonesia	dengue	age specific subgroup	infant/children/ado	hospital	2014	IgG ELISA	3194	69.4	
[139]	Quan TM et al/2017	Vietnam	chik	general population	all ages	previous study	2015	IgG ELISA	546	13.4	
[74]	Ramos MM et al/2008	USA	dengue	general population	all ages	household	2005	IgM+IgG ELISA & MNT	141	39	
[74]	Ramos MM et al/2008	Mexico	dengue	general population	all ages	household	2005	IgM+IgG ELISA & MNT	132	77	
[72]	Ranjan P et al/2016	India	dengue	blood donors	adults	hospital	2012	IgM+IgG ELISA & RTPCR	200	58	
[75]	Reiskind MH et al/2001	Peru	dengue	general population	all ages	household	1996	IgG + PRNT	1225	29.4	
[56]	Rioth M et al/2011	Haiti	dengue	age specific subgroup	infant/children	center recruitment	2007	PRNT	166	65	
[76]	Rodriguez H et al/2005	Colombia	dengue	general population	all ages	household	2004	IgG ELISA	252	23.3	

[155]	Rodriguez-Barraquer I et al/2015	India	dengue+chik	general population	all ages	household	2011	IgG ELISA	1010	93	44	
[164]	Saba Villaroel MS et al/2018	Bolivia	dengue+chik+zika	blood donors	adults	blood donors center	2016	IgG ELISA & Neutralization tests	105	90	46.7	39
[164]	Saba Villaroel MS et al/2018	Bolivia	dengue+chik+zika	blood donors	adults	blood donors center	2017	IgG ELISA & Neutralization tests	200	93.5	54.6	21.5
[152]	Schwarz NG et al/2012	Madagascar	dengue+chik	pregnant women	ado/adult	hospital	2010	IFA & NS1	195	17.4	44.6	
[152]	Schwarz NG et al/2012	Madagascar	dengue+chik	pregnant women	ado/adult	hospital	2010	IFA & NS1	251	10.8	22.7	
[152]	Schwarz NG et al/2012	Madagascar	dengue+chik	pregnant women	ado/adult	hospital	2010	IFA & NS1	395	6.8	2.05	
[152]	Schwarz NG et al/2012	Madagascar	dengue+chik	pregnant women	ado/adult	hospital	2010	IFA & NS1	378	2.25	0.55	
[110]	Seidahmed OM et al/2012	Sudan	dengue	general poulation	all ages	household	2008	IgM ELISA	791	5.2		
[134]	Sergon K et al/2007	Comores	chik	general population	all ages	household	2005	IgM +IgG ELISA	331		63	
[133]	Sergon K et al/2008	Kenya	chik	general population	all ages	household	2004	IgM +IgG ELISA	302		72	
[78]	Shah PS et al/2017	India	dengue	general population	all ages	household	2011	IgG ELISA	855	50.8		
[79]	Sharp TM et al/2015	Angola	dengue	general population	all ages	household	2013	IgM ELISA & RT-PCR	455	10		
[80]	Shu PY et al/2002	Taiwan	dengue	general population		household	1997-1998	NS1 & PRNT	1317	41.9		
[81]	Silva-Nunes M et al/2006	Brazil	dengue	general population	all ages	household	2004	IgM ELISA & HI	358	3.9		
[124]	Simmons G et al/2016	Puerto Rico	chik	blood donors	adults	blood donors center	2015	IgM +IgG ELISA	1031		23.5	
[83]	Siqueira-Junior JB et al/2008	Brazil	dengue	general population	all ages	household	2002	EIA	2906	37.3		
[136]	Sissoko D et al/2008	Mayotte	chik	general population	all ages	household	2006	IgM +IgG ELISA	1154		37.2	
[135]	Sissoko D et al/2008	Mayotte	chik	pregnant women	NP	laboratory	2005	IgM+IgG ELISA	316		1.6	
[135]	Sissoko D et al/2008	Mayotte	chik	pregnant women	NP	laboratory	2006	IgM+IgG ELISA	629		26	
[84]	Sissoko D et al/2010	Mayotte	dengue	general population	all ages	household	2006	IgG ELISA	1154	22.7		
[47]	Sun J et al/2012	China	dengue	general population	all ages	household	2009	IgM+IgG ELISA	365	27.9		
[147]	Sutherland LJ et al/2011	Kenya	dengue+chik	age specific subgroup	children/ado	previous study	2000-2004	IFA & PRNT	122	****	20	
[147]	Sutherland LJ et al/2011	Kenya	dengue+chik	pregnant women	ado/adult	previous study	2000-2004	IFA & PRNT	419	*****	37	
[86]	Tam CC et al/2013	Sri Lanka	dengue	age specific subgroup	infant/children	household	2008-2009	IgG ELISA	797	51.4		
[87]	Thai KT et al/2005	Vietnam	dengue	schoolchildren	children/ado	school	2002	IgM +IgG ELISA	961	65.7		
[88]	Tiong V et al/2015	Malaysia	dengue	schoolchildren	children/ado	school	2008-2009	IgG ELISA	1410	11		
[102]	Tissera HA et	Sri Lanka	dengue	age specific subgroup	infant/children	household	2008	IgG ELISA	797	52		

	al/2010										
[89]	Yen TS et al/2016	Sao Tome and Principe	dengue	pregnant women	adults	hospital	2003-2004	IgM+IgG ELISA & IFA & NS1	78	35.9	
[90]	Tuntaprasart W et al/2003	Thailand	dengue	schoolchildren	NP	school	2000-2001	IgM+IgG ELISA	283	71	
[91]	Vairo F et al/2014	Tanzania	dengue	blood donors	adults	blood donors center	2011	IgG ELISA & IFA	500	50.6	
[92]	Vallée J et al/2009	Laos	dengue	general population	adults	household	2006	IgM +IgG ELISA	1990	84.6	
[92]	Vallée J et al/2009	Laos	dengue	general population	infant/children	household	2006	IgM +IgG ELISA	1568	9.4	
[93]	Van Benthem BH et al/2005	Thailand	dengue	general population	all ages	household	2001	IgM ELISA	1750	6.5	
[137]	Vilibic-Cavlek T et al/2015	Croatia	chik	general population	all ages	hospital	2011-2012	IIFT	1008		0.9
[153]	Vongpunsawad S et al/2017	Thailand	dengue+chik	general population	all ages	previous study	2014	IgG ELISA	835	79.2	26.8
[151]	Weller N et al/2014	Tanzania	dengue+chik	general population	all ages	household	2007-2008	IgG ELISA & IIFT	1215		
[94]	Weppelmann TA et al/2017	Haiti	dengue	schoolchildren	children/ado	school	2013	IgG ELISA	476	71.2	
[95]	Wilder-Smith A et al/2004	Singapore	dengue	age specific subgroup	adults	center recruitment	2002	IgG ELISA	298	45	
[95]	Wilder-Smith A et al/2005	Singapore	dengue	age specific subgroup	adults	center recruitment		IgG ELISA & PRNT	164	29.8	
[97]	Wood H et al/2014	Antigua-Barbuda	dengue	pregnant women	NP	hospital	2009-2010	IgG ELISA	442	94.7	
[97]	Wood H et al/2014	St Lucia	dengue	pregnant women	NP	hospital	2009-2010	IgG ELISA	442	97	
[97]	Wood H et al/2014	St Kitts Nevis	dengue	pregnant women	NP	hospital	2009-2010	IgG ELISA	442	100	
[97]	Wood H et al/2014	Montserrat	dengue	pregnant women	NP	hospital	2009-2010	IgG ELISA	442	80	
[97]	Wood H et al/2014	Jamaica	dengue	pregnant women	NP	hospital	2009-2010	IgG ELISA	442	100	
[97]	Wood H et al/2014	Grenada	dengue	pregnant women	NP	hospital	2009-2010	IgG ELISA	442	98	
[97]	Wood H et al/2014	Dominica	dengue	pregnant women	NP	hospital	2009-2010	IgG ELISA	442	98	
[97]	Wood H et al/2014	Bermuda	dengue	pregnant women	NP	hospital	2009-2010	IgG ELISA	442	8	
[97]	Wood H et al/2014	Belize	dengue	pregnant women	NP	hospital	2009-2010	IgG ELISA	442	36	
[97]	Wood H et al/2014	St Vincent Grenadines	dengue	pregnant women	NP	hospital	2009-2010	IgG ELISA	442	94	
[38]	Yap G et al/2013	Singapore	dengue	general population	all ages	household	2007	IgM+IgG ELISA	3939	65.9	
[99]	Yew YW et al/2009	Singapore	dengue	age specific subgroup	adults	previous study	2004	IgM+IgG ELISA	4152	59	
[100]	Velasco-Salas ZI et al/2014	Venezuela	dengue	general population	all ages	household	2010-2011	HI	2002	77.4	

***Seroprevalence (%)**

**** RDT: rapid diagnostic test (in this study: immunochromatographic assay)**
*****Seroprevalence by serotype: DENV-1 (26.1%); DENV-2 (24.1%); DENV-3 (24.9%); DENV-4 (46.3%)**
****** Seroprevalence by serotype: DENV-1 (9%); DENV-2 (7%); DENV-3 (6%); DENV-4 (8%)**
******* Seroprevalence by serotype: DENV-1 (63%); DENV-2 (67%); DENV-3 (55%); DENV-4 (44%)**
